# Comparison of expectations and beliefs about good teaching in an academic day release medical education program: a qualitative study

**DOI:** 10.1186/1472-6920-14-211

**Published:** 2014-10-03

**Authors:** Thea ACM van Roermund, Henk G Mokkink, Ben JAM Bottema, Chris van Weel, Albert JJA Scherpbier

**Affiliations:** Education & Research Department, Fontys University of Applied Sciences, PO Box 347, 5600 AH Eindhoven, The Netherlands; Department of Primary and Community Care, Radboud University Nijmegen Medical Centre, Nijmegen, The Netherlands; Department of Primary and Community Care, Radboud University Nijmegen Medical Centre, Nijmegen, The Netherlands; Department of Primary and Community Care, Radboud University Nijmegen Medical Centre, Nijmegen, The Netherlands; Australian Primary Health Care Research Institute, Australian National University, Canberra, Australia; Faculty of Health, Medicine and Life Sciences, Maastricht University, Maastricht, The Netherlands

**Keywords:** Educational expectations and beliefs, Teachers, Residents, Postgraduate day release program in medical education

## Abstract

**Background:**

In a professional learner-centered(ness) educational environment, communication and alignment of expectations about teaching are indispensable. Professional education of residents could benefit from an analysis and comparison of teachers’ and residents’ educational expectations and beliefs. Our purpose is to identify success factors and barriers related to aligning expectations and beliefs and building a supportive professional learner-centered educational environment.

**Methods:**

We conducted semi-structured individual interviews with teachers and semi-structured focus groups with residents. A single interview format was used to make it possible to compare the results. Data were analysed using a qualitative software package (AtlasTi). Data analysis steps were followed by the author team, which identified four domains of good teaching: personal traits, knowledge, relationships and teaching qualities.

**Results:**

Teachers and residents agreed about the importance of personal professional characteristics like being a role model and having an open and enthusiastic attitude. They all thought that having a specific knowledge base was essential for teaching. Approaching residents as adult learners was found to be an important element of the learner-centred environment and it was agreed that teachers should take practical experiences to a higher level. However, teachers and residents had different expectations about the practical consequences of being a role model, adult learning, coaching and openness, and the type of knowledge that was needed in the professional development program. Communication about different expectations appeared to be difficult.

**Conclusions:**

Teachers and residents agreed on a conceptual level about expectations and beliefs regarding good teaching, but disagreed on an executive level. According to the residents, the disagreement about good teaching was not the biggest barrier to creating alignment and a supportive professional relationship; instead, it was the absence of a proper dialogue regarding issues about expectations and beliefs.

**Electronic supplementary material:**

The online version of this article (doi:10.1186/1472-6920-14-211) contains supplementary material, which is available to authorized users.

## Background

Under the influence of current educational theories, learner-centeredness has become the main focus of postgraduate medical education in the past 20 years [1–7]. In this learner-centered environment, residents are expected to actively take responsibility for their learning processes, make learning plans and express their learning needs to the teachers [8–11]. Because the learning plans and needs should be aligned with the program’s educational goals and the way teachers prefer to teach, it is important that residents and teachers be able to exchange expectations and beliefs about teaching. When these expectations and beliefs are not compatible, communication about differences should be possible [1, 12, 13]. Medical education research that compares teachers’ expectations and beliefs with those of residents could be helpful in creating a supportive learner-centered environment by showing factors of success and barriers for teaching.

The majority of training of residents takes place in practice. In some countries, training is supported by a program outside practice called the academic day release program, in which reflection on practical experiences in small group sessions is an important element [14–16]. Because the main purpose of the program is to support residents in learning and applying their knowledge in practice, alignment between teachers and residents is extremely important.

Teachers in the academic day release program are experienced doctors, and professionals who are otherwise engaged in health care, such as psychologists. They know how to act in a doctor-patient relationship or a psychologist-patient relationship and have experience in training the individual residents in their practices. However, they may have little experience with teaching groups of residents [14–16].

During their time in the academic release program, teachers discover that the concept of learner-centeredness means that they should balance teaching a group, the curriculum’s purposes, the residents’ expectations and beliefs about teaching, and their own personal beliefs about teaching [10, 17]. In their turn, residents should balance asking for feedback on their performance, expressing their learning needs and discussing their expectations about teaching with their teachers [6, 18–21]. Incongruities in this interaction process between the teacher and the resident do not necessarily pose a problem as long as both parties are able and encouraged to face and reflect on expectations and beliefs [22].

Studies that compare teachers’ and learners’ beliefs and expectations usually concern teaching in elementary and secondary schools [23–27]. As far as we know, research about educational expectations and beliefs in higher education has focused on either teachers or learners, but little attention has been paid to them both in formal learning sessions [24]. In this study, we compare teachers’ and residents’ beliefs in an academic day release program, in order to identify similarities that could support and differences that could hinder teaching in the learner-centered environment.

The study was conducted in the Netherlands, in the postgraduate program for General Practitioners (GPs), offered by the Departments for General Practice Training of the eight university medical centres. The program comprises two years of training in general practice, interspersed with one year in hospital and community services. Residents meet at their university’s medical department one day per week to participate in the academic day release program. Small groups of 10 to 12 residents are coached by two teachers, including an experienced general practitioner and a behavioural scientist. They are both responsible for teaching the program as well as for coaching the professional development and for judging the performance and development of the residents.

Our research questions are:

1. What are teachers’ and residents’ expectations and beliefs about what and how to teach in an academic day release program?
2. Do they agree about what and how to teach?
3. Do teachers and residents communicate about what and how to teach in order to support a learner-centered environment?

## Methods

### Design

We conducted semi-structured individual interviews with teachers and semi-structured focus groups with residents. The transcripts of the interviews and focus groups were analysed separately. To enable the comparison of results, the same interview format was used for both teachers and residents (see Additional file 1).

The research method used complies with the Qualitative research review guidelines – RATS.

### Ethical approval

This study was carried out in the Netherlands in accordance with the applicable rules concerning the review of research ethics committees and informed consent (The Research Ethics Committee of the Radboud University Nijmegen Medical Centre, file number CMO 2012/412).

### Participants

This study involved teachers in the postgraduate academic day release programs for general practitioners and their resident groups.

### Inclusion criteria

For the individual interviews, we invited teachers (general practitioners and psychologists) who had worked with the same group of residents in weekly sessions for at least three months. For the focus groups, we aimed to include only groups of at least five to six residents to ensure variety in their views. In order to be able to answer the question about communication, we decided to include teachers and resident groups who actually worked with each other.

### Data collection procedure

Teachers were informed collectively about the interviews and invited for individual interviews with one of the researchers (TR). The interviews were conducted in the department for training of general practitioners in two university medical centres and lasted for 45 to 60 minutes. All interviews were recorded and transcribed verbatim.

Teachers asked resident groups to participate and then one of the researchers (TR) informed the resident groups about the study and the interviews. All interviews lasted for 60 to 75 minutes and were recorded and transcribed verbatim.

Two departments were involved in the data collection: we interviewed 12 teachers and 7 resident groups from the first department and 5 teachers and 2 resident groups from the second department. After each individual interview and focus group, we checked whether new information had emerged. Interviewing continued until saturation was reached, which occurred after interviewing people from the second department.

### Data analysis: development of dimensions

In accordance with Miles and Huberman’s theory about qualitative data analysis [28], three consecutive phases were used for the individual and focus group interviews: data reduction by coding, data structuring by categorization and data interpretation by discussion. On the basis of this analysis, we developed a framework for comparison [29].

TR imported all interview transcripts into the AtlasTi software package and coded all items. The codes were used as a first coding dictionary. Two researchers (TR and HM) revised the coding dictionary together by removing code duplicates and discussing the codes until they reached consensus about covering the items. For data reduction, these researchers used the tree structure from the AtlasTi software package. They independently structured the codes and discussed their structures in order to identify dimensions of teaching. Four underlying dimensions were identified: (1) personal traits, (2) knowledge/expertise, (3) relationships/ communication and (4) teaching qualities.

To test the accuracy of the dimensions, BB, CW and AS structured a random sample of 45 items independently. Cohen’s κ was calculated for inter-rater reliability. Inter-rater reliability is the degree of agreement among raters. To test the accuracy, according to topical literature, a Cohen’s κ of .70 is acceptable [30]. Inter- rater agreement was .73-.76, which is sufficient.

Data interpretation by discussion was the connecting activity throughout the whole analysis process and during the decision-making process about relevant quotes.

### Data analysis: comparison

To compare the teachers and residents, we used all relevant data that were connected to the four dimensions. The first analysis procedure (coding and structuring) provided a practical tool for this task. The data were summarized independently per dimension after discussion between the authors. An example of this procedure is shown in Table 1; it regards dimension 1: personal traits. After that, characteristic quotes were chosen to illustrate the findings.

**Table 1:** **Example of data analysis**

Personal traits by teachers	Personal traits by residents
***Relevant data***	***Relevant data***
-*Liking my job as a doctor is essential (GP1)*	-*We like a teacher who shows that he likes to teach (R8)*
-*For me it is important that a doctor is able to show vulnerability (GP2)*	-*Teachers should be open and critical about themselves (R1)*
-*I think humour is important and having fun during day release time is too (GP3)*	-*Things work out better in our group when a teacher is determined and powerful (R2)*
-*I teach the residents enthusiasm for the medical profession (GP4)*	-*We need an enthusiastic teacher who can communicate clearly (R6)*

## Results

Table 2 presents the characteristics of the participants in the two departments for general practitioners’ training.

**Table 2:** **Characteristics of the participants**

Teachers (N = 17)		Residents (N = 100, 9 groups)	
Characteristic	Description	Characteristic	Description
Men	10	Men	19
Women	7	Women	81
General practitioners	9	First-year trainees	4 groups
Behavioural scientists	8	Second-year trainees	2 groups
Age	30-60 years	Third-year trainees	3 groups
Work experience	1-20 years	Age	25-45 years

In this section, we will first describe teachers’ and residents’ beliefs and expectations for each of the four dimensions: personal traits, knowledge, relationships and teaching qualities. Next, we will compare these expectations and beliefs to identify factors of success for and barriers to teaching in a learner-centered environment.

In the personal dimension, we found that teachers see ‘openness’ as revealing one’s strengths and weaknesses, and they expect the residents to do the same. As a consequence, they strongly believe that residents should learn to deal with their own vulnerabilities by talking freely about their mistakes. For residents, openness means that teachers give transparent feedback and are willing to share their professional experience with them. They primarily see the teacher as a role model with professional strengths and leadership (Table 3).

**Table 3:** **Personal traits dimension**

	Teachers	Residents
Quotes	*“Enthusiasm is what you really need in this work.” (P-GP8)*	*“Being enthusiastic is the main thing.” (P-R2)*
	*“Being a role model and being able to show one's vulnerability is one of the most important qualities here. A doctor should be able to talk openly about his mistakes.” (P-GP2)*	*“The teacher should be willing to show leadership and talk about his experiences as a role model.” (P-R5).*
		*“Teachers should be transparent in giving feedback.” (R-R3)*

As for knowledge, teachers thought they could rely on the knowledge they have gained from practical experience for teaching less experienced junior doctors. Their interpretation of relevant teaching knowledge is the knowledge of group dynamics and they viewed that as crucial to their teaching roles. The residents interpreted knowledge as medical expert knowledge, which would enable discussions about residents’ experiences with difficult situations to be raised to a higher level (Table 4).

**Table 4:** **Knowledge/expertise dimension**

	Teachers	Residents
Quotes	*“I don’t need to know all the ins and outs of cardiac problems. My daily practical knowledge is sufficient for working with residents.” (K-GP4)*	*“Teachers should be able to explain theoretical considerations underlying treatments and discuss the limitations of guidelines.” (K-R4)*
	*“Group dynamics: that is what you have to learn about when you start working as a teacher. Because that is difficult to handle.” (K-P2)*	*“The psychologist teacher should have higher-level knowledge about doctor-patient communication and how to deal with conflict.” (K-R8)*

Regarding the dimension of communication and relationships, teachers were inclined to take a caring role. Although the teachers saw residents as adults and built an atmosphere of confidence, they thought they knew best. To the residents, confidence meant being able to talk freely about what they expect from their teachers. They admitted to occasional immature behavior, but they wanted their teachers to discuss mutual expectations with them on an equal basis and they also felt that teachers should stimulate and initiate an open exchange of views.

Coaching and giving support while simultaneously evaluating the residents’ professional development were indicated to be conflicting roles because confidentiality is an important element of the educational atmosphere (Table 5).

**Table 5:** **Relationships/communication dimension**

	Teachers	Residents
Quotes	*“Residents are adult learners; however, as teachers, we take care and know what is best for them, although they sometimes disagree.” (R-GP6)*	*“We want to be treated as adults, although we do not always act that way.” (R-R6)*
	*“Besides providing guidance and support, I also have to give feedback on their professional development and these are conflicting roles.” (R-GP1)*	*“Confidentiality is an important issue here, because coaching and evaluation are in the hands of the same teachers.” (R-R8)*
	*“If something happens in the group and the atmosphere is getting worse, then I have to pick up some problematic issues in the group dynamics.” (R-P3)*	*“I think a lot of energy is wasted because we hardly ever talk explicitly about our expectations regarding certain issues. Teachers should stimulate us to do so, but our feedback is not always welcome.” (R-R7)*

Regarding the teaching dimension, teachers interpreted their coaching role as ‘counselling’ in terms of listening to residents’ stories and encouraging them to examine what these stories meant to them in order to better understand their experiences. Residents interpreted teaching competence as being able to explain connections between theoretical and practical knowledge, being proactive and delivering a program that is on a par with continuing professional development programs for more experienced colleagues (Table 6).

**Table 6:** **Teaching dimension**

	Teachers	Residents
Quotes	*“My inspiration comes from helping residents to analyse their experiences in relation to personal growth.” (T-P3)*	*“He should be able to make the translation from science to practice.” (T-R1)*
	*“I learned to ask: what does this experience mean to your personal development from a counselling perspective?” (T-GP1)*	*“I learned a lot from well-organized continuing education programs.” (T-R9)*
	*“My mission is to show residents that a patient is a human being, not just a disease that has to be cured.” (T-P6)*	*“We appreciate a proactive way of teaching. Teachers who take teaching us as seriously as teaching experienced colleagues.” (T-R6)*

### Comparison

The results show that teachers and residents agreed about the importance of a teacher being a role model and having personal characteristics such as enthusiasm and openness. They also agreed that teachers should have a certain knowledge base and should treat residents as adult learners.

However, teachers and residents also had different expectations and beliefs in each of the dimensions. They did not agree about who should be open about what, the way residents should be treated, what kind of knowledge teachers should have and how this knowledge should be transmitted.

Regarding the third research question about communication between teachers and residents, we found that while teachers thought that they picked up issues in the residents group, residents stated that they hardly ever explicitly discussed beliefs and expectations. Residents thought that teachers should stimulate and initiate an open exchange of views. But when they took the initiative to give feedback, their feedback was not always welcome and residents felt like they were wasting their energy.

## Discussion

In this study, we collected teachers’ and residents’ expectations and beliefs about teaching in the postgraduate academic day release program for general practitioners in the Netherlands. We then compared these beliefs and expectations in order to identify factors of success and barriers to a professional learner-centered environment. We found four dimensions of teaching that covered the expectations and beliefs: personal traits, knowledge/expertise, relationships/communication and teaching qualities.

General agreement was found in each of the dimensions:

– the teacher should be a role model, be enthusiastic and have an open attitude
– the teacher should have a certain knowledge base
– the teacher should acknowledge that residents are adult learners
– coaching and assessing are conflicting roles

Teachers and residents did not agree about how these beliefs and expectations should be made effective (i.e. which themes they should be open about, what kinds of knowledge should be conveyed by teachers, how teachers ought to act as coaches for adult learners and how they should deal with various teaching roles). To summarize, teachers and residents agreed at a conceptual level about expectations and beliefs regarding good teaching, but disagreed at an executive level.

Agreement between teachers and residents about their expectations and beliefs can be a factor of success for a learner-centered environment. In current research about the evaluation of teaching performance, Scheepers et al. [31] showed that teachers who have an ‘agreeable professional attitude’ are able to consciously discuss residents’ learning needs and educational goals. These teachers are flexible when it comes to adapting residents’ learning plans and needs to the goals of the educational program. Moreover, they point to residents’ responsibilities with regard to learning.

Disagreement itself does not need to be a barrier to communication between teachers and residents in an academic day release program, but it can make it more difficult to align the residents’ learning needs and plans to the educational purposes of the program. Researchers in communication identified communication and feedback as one of the most important issues affecting teacher-resident interaction that should enhance residents’ professional growth [9, 32, 33]. The degree to which educational goals are achieved depends on both the teacher’s and learner’s abilities to negotiate with one another and resolve conflicts [32]. The residents in our study experienced the absence of a real dialogue that would contribute to aligning expectations and beliefs, while teachers seemed to be convinced that they brought up issues in the group and, moreover, that they knew what was best for the residents. Teachers seemed to take a paternalistic perspective in communication with residents [34]. This paternalistic attitude was common in medical practice from its ancient roots until the 1970s, when doctors came to see that professional paternalism towards patients is ethically unacceptable and that they should respect the patients’ autonomy and informed consent [35]. In a paternalistic educational climate, teachers are likely to take over learning responsibilities from their residents instead of stimulating the residents to face and deal with these responsibilities. The absence of a real dialogue in the academic day release program can be a serious barrier to aligning expectations, beliefs and professional support.

Another possible barrier for a professional learner-centred environment in the educational model of the GP training program seemed to be the educational model. From current educational theories about the learner’s responsibility for his learning process, one could assume that a learner-centred environment seems to offer the best climate for a dialogue about expectations and beliefs. In his meta-analysis, Cornelius-White [1] explained that learner-centred education is a model that originated in counselling and was based on the client-centred approach founded by Carl Rogers [36]. In this approach, a counsellor invites the client to speak freely about his or her experiences, desires and worries. According to McCombs [4] and Bingham and Sidorkin [37], empathy and honouring the learner’s voice are key elements in a corresponding educational approach. The teachers in our study interpreted teaching as ‘counselling’ in correspondence with the psychological humanistic perspective and a modern educational concept of learner- centeredness. However, they seemed to ignore the residents’ expectations that they would show leadership and expert knowledge and be proactive.

A third barrier could be the academic day release context itself. More specifically, the synergy of two teachers teaching one group of residents, could strengthen a barrier that keeps residents from expressing their expectations and learning needs. Research about co-teaching in higher education (i.e. two teachers, who have different specialties, teaching in one group of students), indicates that it is important for the students’ learning process to be transparent when teachers have different beliefs about what and how to teach [38–40]. When teachers are open about differences, students learn how to use differences as a source of discussion about their learning goals and needs [41, 42]. In our study, teachers themselves strongly agreed about their teaching roles as counsellors and, together, they confirmed each other in these roles as the best fit for supporting learning in practice. On the basis of this confirmation, it is plausible that doctors and psychologists strengthened their own and each other’s beliefs about teaching: “as teachers, we know what is best”. As a result, residents got the feeling that “feedback is not welcome”.

Finally, the struggle with teaching roles (counselling and assessing) in which confidence and confidentiality are essential, could hinder an open exchange of expectations in the professional learning environment. The teachers’ goals were to contribute to the personal growth of residents and, accordingly, they believed that counselling was the most important role. According to Bandura [43], Parker et al [44] and DeShon and Gillespie [45], self-goals and increasing agency (i.e. personal control over one’s environment) are the most fundamental aspects of human existence. Failure to establish agency is believed to be strongly related to poor self-esteem. In this psychological perspective it is likely that teachers tend to hold on to their beliefs instead of aligning these with the expectations of the residents and the educational program goals.

## Conclusion

With respect to the four teacher characteristics personal traits, knowledge/expertise, relationships/communication and teaching qualities, teachers and residents agreed on a conceptual level about expectations and beliefs, but disagreed on an executive level. According to the residents, the disagreement about good teaching was not the biggest barrier to creating alignment and a supportive professional relationship; instead, it was the absence of a proper dialogue regarding issues about expectations and beliefs. Other barriers were the educational model of the GP training program, the academic day release context and the struggle with teaching roles.

### Future research

Questions for further research could focus on what ‘learner-centeredness’ means in an academic day release learning environment that aims to support learning in practice, and on the conditions for communication about differences between teachers’ and residents’ expectations and beliefs about teaching. Research in this specific educational context could explore the effects of co-teaching.

Future research could also focus on how these themes can be investigated (i.e. what research methods are most suitable for searching for answers). One option is an organizational learning history method that focuses on the various perspectives of teachers and residents, starting with collecting and then selecting critical events that can be reflected upon.

### Strengths and limitations

To our knowledge, this is one of the rare qualitative studies to critically compare teachers’ and residents’ beliefs and expectations about teaching roles in academic day release training. The study also offers insights into possible consequences of lack of communication about teaching in a learner-centered educational environment (e.g. how teachers can interpret their role as coach).

We should, however, also point out some limitations. The study was limited to the training of general practitioners in the Netherlands, in which day release training plays an important role. These Dutch programs are representative for formal learning programs, but comparable long-term relationships between teacher and resident groups in postgraduate formal training are not common throughout the world. Although our results are drawn from a local context, we think they can inform the work in other formal medical educational environments.

A second point of interest could be the complexity of the qualitative comparison methodology. We followed up on this complexity by conscientiously conducting and discussing the subsequent research steps with an experienced team of researchers.

## Electronic supplementary material

Additional file 1: **Interview format.** (DOC 30 KB)
