# Supplementary material for: Comparison of expectations and beliefs about good teaching in an academic day release medical education program: a qualitative study
Source: BMC Med Educ. 2014 Oct 3;14:211. doi: 10.1186/1472-6920-14-211 (PMC4198618; doi:10.1186/1472-6920-14-211)
Supplement: Supplementary file 1 — Additional file 1: Interview format. (DOC 30 KB) [file 12909_2013_1037_MOESM1_ESM.doc]

**Appendix 1 Interview format**

| Teacher nr/ Group nr | Date | Notes |
| --- | --- | --- |
| Themes | Subthemes |  |
| 1. Expectations and beliefs | - What were your expectations when you arrived in the program? - Did reality match these expectations? - What did you do when expectations and reality did not correspond? |  |
| 1. Motivations and beliefs | - In your opinion, what are important conditions for participating as a teacher/ resident? - What are the barriers to realizing an effective academic day release training program? |  |
| 1. Important teaching characteristics | - What should teachers know and be able to do to create a good learning environment? - How should teachers and residents interact? |  |
